# Modulating *ilvA* encoding threonine deaminase for balanced growth and PHB synthesis by *Halomonas* grown in rich nitrogen source

**DOI:** 10.1016/j.synbio.2026.03.022

**Published:** 2026-04-16

**Authors:** Junting Sheng, Yuying Guan, Jiale Wang, Xu Liu, Zhenghao Xu, Yiling Chen, Fuqing Wu, Guo-Qiang Chen

**Affiliations:** a School of Life Sciences, Tsinghua University, Beijing, 100084, China; b Center for Synthetic and Systems Biology, Tsinghua University, Beijing, 100084, China; c Tsinghua-Peking Center for Life Sciences, Beijing, China; d MOE Key Lab of Industrial Biocatalysis, Dept Chemical Engineering, Tsinghua University, Beijing, 100084, China; e State Key Laboratory for Biomanufacturing, Tsinghua University, Beijing, 100084, China; f State Key Laboratory of Animal Nutrition and Feeding, Institute of Animal Sciences, Chinese Academy of Agricultural Sciences, Beijing, 100193, China

**Keywords:** *Halomonas*, PHB, *ilvA*, sRNA, SspB, ClpXP, Nitrogen rich condition, Next generation industrial biotechnology, NGIB

## Abstract

Bioplastic poly(3-hydroxybutyrate) (PHB) production by *Halomonas bluephagenesis* is typically activated under nitrogen limitation, inevitably restricting biomass formation and overall productivity. Here we identified gene *ilvA* encoding threonine deaminase as a flux-sensitive node linking branched-chain amino-acid synthesis to nitrogen sensing. Complete *ilvA* deletion or σ^54^ disruption in *H. bluephagenesis* created a pseudo-nitrogen-limitation state that increased PHB accumulation yet concurrently suppressed microbial growth. To overcome this trade-off, two independent *ilvA* fine-tuning strategies were evaluated including synthetic sRNA-mediated translational repression and SspB/ClpXP proteolysis. In contrast, complete *ilvA* deletion created a pseudo-nitrogen-limited state that elevated PHB synthesis however reduced cell growth (cell dry weight or CDW) in 7 L bioreactors, reaching only 60 g/L CDW containing 80% PHB, far below the wild-type grown to 95 g/L CDW containing 60% PHB under same conditions, These results indicate that complete deletion of *ilvA* disrupts the balance between cell growth and PHB production. Under nitrogen rich fed-batch conditions, however, sRNA-based partial repression maintained CDW at 95 g/L while increasing PHB from 60% to 80 wt%, thereby establishing a growth-PHB production balance. These results suggest that controllable attenuation of *ilvA*, instead of full gene deletion, provides a potentially scalable approach for improving PHB production without severely compromising cell growth.

## 1 Introduction

Polyhydroxyalkanoates (PHAs) are intracellular polyesters that many bacteria synthesize to buffer imbalances in carbon and redox supply [1]. Among them, poly(3-hydroxybutyrate) (PHB) has gained wide industrial interest due to its biosynthetic simplicity, biodegradability and material properties [2,3]. In many fed-batch growth processes, PHB accumulation is triggered by nitrogen limitation, which increases the allocation of acetyl-CoA and NADPH to polymer synthesis but concurrently suppresses biomass formation [4,5].

Consistent with this paradigm, previous studies have generally reported that nitrogen-rich conditions favor biomass formation at the expense of PHB accumulation, as carbon flux is preferentially directed toward growth rather than storage polymers under nutrient sufficiency [6,7]. For example, *Alcaligenes latus* accumulated substantially higher PHB under nitrogen limitation than under nitrogen-sufficient conditions [8], and broader surveys across diverse microorganisms have likewise shown that PHB yields are typically enhanced when nitrogen is limiting rather than abundant [6].

At the mechanistic level, PHB synthesis is regulated at multiple layers, including carbon flux distribution, nitrogen metabolism, key enzyme expression, and cellular physiological state [7,[9], [10], [11]]. More broadly, recent advances in metabolic engineering have emphasized the potential of dynamically regulating key metabolic nodes to mitigate the intrinsic trade-off between cell growth and product formation in microbial production systems [12,13]. Consequently, manipulating polymer accumulation without invoking nutrient limitation remains challenging, as perturbations at any single layer often propagate through tightly coupled metabolic and regulatory networks [10].

*Halomonas bluephagenesis*, a halophilic and alkaliphilic microbial chassis that supports low-cost, open and continuous bioprocessing, has been widely explored for productions of PHB and other chemicals [[14], [15], [16]]. Nevertheless, achieving high PHB accumulation under nitrogen-rich conditions remains a central challenge. Nitrogen sufficiency generally prioritizes protein biosynthesis and growth over storage-polymer PHB formation [17], suppressing PHB titers even in strains engineered for high polymer capacity. Previous efforts to improve PHB under non-limiting nitrogen have focused mainly on blocking intracellular depolymerization for example via deletions on PHB depolymerases encoded by *phaZ*s [18] or strengthening *de novo* synthesis. However, in *H. bluephagenesis*, *phaZ* deletions failed to raise PHB synthesis when urea was abundant, suggesting that polymer turnover is not the primary bottleneck under nitrogen-rich conditions.

Instead, whole-genome sequencing of a high-PHB and slow-growth (low CDW) *H. bluephagenesis* mutant unexpectedly revealed mutations in *ilvA* encoding threonine deaminase as the entry step to branched-chain amino-acid (BCAA) biosynthesis, similar to *rpoN* (σ^54^), a master regulator of nitrogen utilization [19,20]. Perturbing both genes reproduced a convergent “low-CDW/high-PHB” phenotype, implicating BCAA entry flux and σ^54^-dependent nitrogen signaling may influence carbon-nitrogen partitioning under nitrogen sufficiency. Threonine deaminase (*IlvA*) catalyzes the conversion of l-threonine to 2-ketobutyrate and ammonia, providing the direct precursor for isoleucine biosynthesis and shaping the balance between amino-acid supply, acetyl-CoA partitioning and nitrogen assimilation [21]. Because the reaction is feedback-inhibited by isoleucine [22], the enzyme functions as a key control point at the entry of the branched-chain amino acid biosynthetic pathway. Consequently, changes in *IlvA* activity can influence intracellular nitrogen status and alter the partitioning of carbon flux between cellular growth and PHB synthesis. Motivated by these observations, it is of interest to determine whether fine-tuning *ilvA* expression could expose a growth-compatible regime that enables PHB accumulation under nitrogen-rich conditions. To achieve finer-scale and multi-layer regulation in this study, small regulatory RNAs (sRNAs) [23] and rapid adjustment of protein levels by SspB/ClpXP based protein degradation [24] were investigated for post-translational control, aiming to offer speed, reversibility and dynamic control of balancing cell growth and PHB production.

## 2 Methods

### 2.1 Plasmids, strains and culture media

All plasmids and strains constructed in this study are listed in Table s1 and s2, All DNA fragments were PCR-amplified with Phanta Super-Fidelity DNA Polymerase (Vazyme). All editing cassettes and expression constructs were assembled in plasmids derived from the modular pSEVA321 or 341 [25], which served alternately as recombineering vehicles and heterologous-expression backbones. Plasmid construction was carried out using the Gibson Assembly protocol [26].

*Halomonas bluephagenesis* [27] was grown at 37 °C and 200 rpm in 60LB (60 g/L NaCl, 10 g/L tryptone, 5 g/L yeast extract). *Escherichia coli* S17-1 was cultured under the same temperature and shaking speed in 10LB (10 g/L NaCl, 10 g/L tryptone, 5 g/L yeast extract).

For PHA synthesis, *H. bluephagenesis* was transferred to mineral medium 60 (60MM) composed of 60 g/L NaCl, 1 g/L yeast extract, 0.2 g/L MgSO_4_, 0.5 or 1 g/L urea, 10 g/L Na_2_HPO_4_·12H_2_O, 1.5 g/L KH_2_PO_4_, plus 10 g/L trace-element solution I and 1 g/L trace-element solution II [28]. Seed cultures were generated in two successive stages. First, a single colony of wildtype *Halomonas bluephagenesis* TD1.0 (or its derivatives) was grown overnight at 37 °C in 20 mL of 60LB in a 200 mL Erlenmeyer flask, forming the primary seed. Next, 200 μL of this overnight culture was transferred to a fresh 20 mL 60LB in the same flask and incubated for a further 10 h at 37 °C, producing the secondary seed used for subsequent experiments. Cultures were incubated at 37 °C. 60MM was set to pH 8.5 with NaOH before sterilization. Cultures were incubated for 48 h at 37 °C while shaking at 200 rpm, and each condition was set in biological triplicate.

When required, antibiotics were added at the following final concentrations: kanamycin 50 μg mL^−1^, chloramphenicol 25 μg mL^−1^, and spectinomycin 100 μg mL^−1^. Genomic engineering in *Halomonas bluephagenesis* was introduced by conjugation from the donor *Escherichia coli* S17-1 *λpir*.

### 2.2 Conjugation for gene editing

The donor *E. coli* S17-1 carrying the recombinant plasmid, and the recipient *Halomonas bluephagenesis* culture were each grown to mid-log phase (OD600 ≈ 0.4). Cells were collected by centrifugation, rinsed once with 20LB (20 g/L NaCl) to remove residual medium, and the pellets were combined and resuspended in 50 μL fresh 20LB. The mixture was then spotted onto an 20LB agar plate and incubated 6–8 h at 37 °C to permit plasmid transfer.

The next day, the conjugation lawn was scraped into 60LB, and spread onto LB60 agar supplemented with the appropriate antibiotic(s). After 24 h at 37 °C, discrete colonies appeared; these putative colonies were screened by colony PCR to confirm successful plasmid introduction.

### 2.3 Flow cytometry

Bacterial suspensions were diluted by mixing 10 μL of culture with 240 μL phosphate-buffered saline (PBS). From this mixture, 20 μL was loaded into an LSRFortessa-4 flow cytometer (BD, USA). Green fluorescent protein (GFP) was excited with a 488 nm laser, and the emission was captured through the FITC filter set with a photomultiplier voltage of 420 V.

### 2.4 Transmission electron microscopy (TEM)

Ultrathin sections of bacterial cells were examined by transmission electron microscopy (Hitachi H–7650B, Japan) to visualize intracellular PHB storage. 1 mL cell culture was pelleted at 5000×*g* for 1 min, and the supernatant was discarded. Then, immediately suspended in 2.5% (v/v) glutaraldehyde and held at 4 °C for 4 h. Sections were mounted on copper grids and observed at 80 kV. Electron micrographs provided a clear view of PHB granule morphology and distribution within the cytoplasm, enabling an intuitive assessment of storage-polymer accumulation.

### 2.5 CDW and PHB analysis

Cell pellets were obtained from 30 mL culture aliquots by centrifuging at 10000×*g* for 15 min. After a single rinse with 20 mL deionized water, the biomass was taken from frozen storage at −80 °C (1–2 h) and subsequently freeze-dried for ≥12 h to determine cell dry weight (CDW).

Approximately 30−40 mg of the dried cells was subjected to methylation analysis to release intracellular PHB [28]. Samples were mixed with 2 mL methanol containing 3% (v/v) concentrated H_2_SO_4_ and 1 g/L benzoic acid, together with 2 mL chloroform, and heated at 100 °C for 4 h. After cooling to room temperature, 1 mL distilled water was added, the tubes were vigorously vortexed, and the phases were allowed to separate. The chloroform (lower) layer was collected for gas-chromatographic analysis.

Highly purified poly(3-hydroxybutyrate) was processed in parallel as calibration standards. PHB content and monomer composition were determined on a Shimadzu GC-2014 equipped with a flame-ionization detector and a 30 m HP-5 capillary column, following established procedures [29].

### 2.6 Quantitative analyses via high-performance liquid chromatography (HPLC)

Culture broths were clarified by centrifugation at 10,000×*g* for 10 min; the filtrates were injected into a Shimadzu HPLC equipped with a Bio-Rad Aminex HPX-87H column held at 50 °C and eluted isostatically with 5 mM H_2_SO_4_ at 0.60 mL min^−1^. Eluting compounds were detected by UV absorbance at 260 nm. Quantitation employed external calibration: five concentration points were prepared for each reference compound, peak areas were fitted by linear regression (R^2^ > 0.99), and sample concentrations were obtained from the resulting equations [30].

### 2.7 Extraction of amino acids from bacterial cells

The bacterial extraction protocol involves aspirating the medium completely and washing the cells gently three times with PBS buffer, ensuring the buffer is fully aspirated. The cells are then placed on dry ice and treated with 2 mL of 80% methanol, pre-chilled to −80 °C, followed by cell disruption via sonication or grinding. The lysate was incubated at −80 °C for 2 h or overnight, then centrifuged at 14,000×*g* for 20 min at 4–8 °C. The metabolite-containing supernatant was transferred to a new 1.5 mL tube on dry ice, avoiding touching the pellet. The supernatant was lyophilization dried using SpeedVac (SPD120, Thermo Fisher Scientific, USA), or nitrogen without heating at room temperature. If the liquid volume was reduced by half, vortexing was conducted for 10 s. Multiple test tubes were combined during the drying process. The dried samples were stored at −80 °C. The samples must be sent in dry ice as soon as possible after preparation. Important notes include not vortexing for more than 1 min, ensuring sufficient centrifugation time, and avoiding touching the pellets during the transfer of the supernatant. The dried extracts were stored at −80 °C and shipped on dry ice for metabolomic analysis. Metabolomic profiling was conducted by the Tsinghua University Metabolomics Platform (THU-metabolomics) using standardized analytical and data-processing procedures described by the facility.

### 2.8 PHB molecular weight studies via gel permeation chromatography (GPC)

The molecular weights of the PHB samples were determined using gel permeation chromatography (GPC) on a LC-20AD system (SHIMADZU, Japan) equipped with a SHIMADZU GPC-804C column and a refractive index detector (SHIMADZU, Japan). The PHA samples were dissolved in chromatographically pure chloroform at a concentration of 2 mg/mL, followed by filtration through a 0.22 μm nylon syringe filter (Jinglong, China) to eliminate any undissolved particles. Chloroform, of chromatographic grade, served as the mobile phase at a flow rate of 1 mL/min and a column temperature of 40 °C. The injection volume was set at 40 μL. A calibration curve was created using polystyrene standards with varying number-average molar masses (1 × 10^4^, 2 × 10^4^, 3 × 10^4^, 7 × 10^4^, 1.5 × 10^5^, 3 × 10^5^, 7 × 10^5^, and 1 × 10^6^; Sigma-Aldrich, U.S.A).

### 2.9 RNA sequencing

Bacterial samples were collected from shake-flask cultures after 12 h of incubation. The samples were then centrifuged at 4000×*g* for 10 min at 4 °C, followed by rapid freezing in liquid nitrogen. For RNA sequencing and subsequent analysis, the samples were immediately stored on dry ice for analysis. RNA extraction, sequencing and analysis were carried out by Hangzhou Lianchuan Bio Technologies Co. Ltd. In brief, RNA was extracted using TRIzol® Reagent (Invitrogen, U.S.A.), and the RNA library was prepared using the Illumina TruseqTM RNA sample prep Kit (Illumina, U.S.A.). Sequencing was conducted on the Illumina Hiseq platform, with library processing done using the Truseq SBS Kit v3-HS (Illumina, U.S.A.).

### 2.10 PHB production in a 7 L fermenter under high nitrogen condition

The compositions of the batch and feeding media, together with the operating conditions of the bioreactor, were described as follows: For low-nitrogen cultivations, the batch medium contained 60 g/L NaCl, 2 g/L yeast extract, 20 g/L glucose, 3.3 g/L urea, 1 g/L MgSO_4_, 2.69 g/L Na_2_HPO_4_, 3.3 g/L KH_2_PO_4_, 10 mL/L trace element solution I and 1 mL/L trace element solution II, in accordance with previously published formulations [29]. In this experiment, in the nitrogen rich version of this medium, the urea level in the batch phase was increased to 10 g/L. Seed cultures were first grown in the same medium as used in shake flasks. Before inoculation, the optical density of the seed culture was adjusted to OD_600_ = 3, and 300 mL of this culture (10%, v/v of the final working volume) were inoculated into the bioreactor.

All fed-batch experiments were carried out in a 7 L BioFlo bioreactor (New Brunswick Scientific, USA) that was operated with a working volume of 3 L. Throughout the process, the system was run under open non-sterile conditions. The broth temperature was maintained at 37 °C using an external recirculating chiller (Henan Jinghua Instrument, China). To control pH, an automated pump intermittently delivered 5 M NaOH to the culture to maintain pH at 8.5. Agitation started at 200 rpm. As growth proceeded, the stirring rate was gradually increased until it reached 800 rpm at the maximum. This strategy ensured that the dissolved-oxygen (DO) level remained above 30% of the air-saturated value recorded in an empty vessel. Compressed air was supplied at a constant flow of 3 L/min.

## 3 Results

### 3.1 Effects of *phaZ* deletion on PHB synthesis under high-urea conditions

**Fig. 1:**
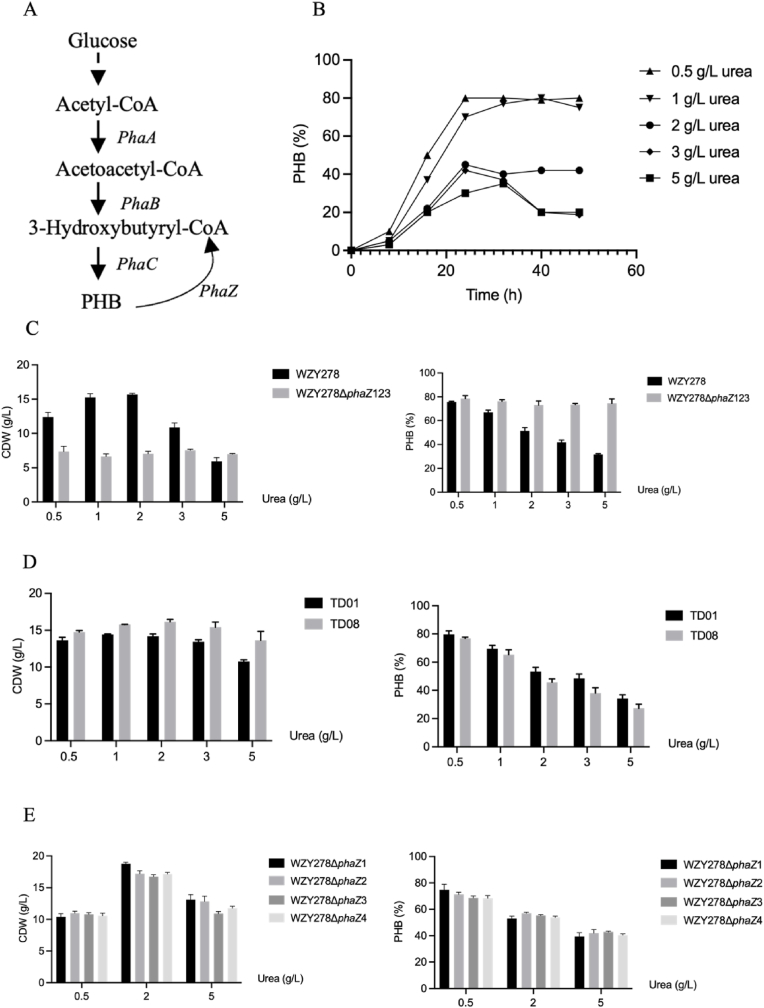
Effects of nitrogen on growth (CDW) and PHB synthesis by *H. bluephagenesis* with or without *phaZ* encoding PHB depolymerases. (A) Metabolic scheme outlining the acetyl-CoA-derived PHB biosynthetic route and the role of PhaZ-mediated intracellular PHB turnover. (B) Time-course PHB accumulation profiles under graded urea concentrations during shake-flask cultivation. (C) Comparative growth (CDW) and PHB production by *H. bluephagenesis* WZY278 and the triple *phaZ*-deleted mutant *H. bluephagenesis* WZY278Δ*phaZ*123 grown across different nitrogen levels, respectively. (D) Physiological comparison of wildtype *H. bluephagenesis* TD01 and TD08 (knock out three *phaZ* genes by CRISPR/Cas9) grown under various urea concentrations, respectively, measured using CDW and PHB content. (E) Evaluation of CDW and PHB accumulation by four independent *H. bluephagenesis* WZY278 *phaZ*-deleted isolates grown under different nitrogen conditions, respectively.

The PHB biosynthetic pathway in *H. bluephagenesis* proceeds from glucose-derived acetyl-CoA, which is condensed by PhaA, reduced by PhaB, and polymerized by PhaC into PHB, while intracellular depolymerization is mediated by *phaZ* encoding PHB depolymerase (Fig. 1A). At low urea concentrations (0.5–1 g/L), PHB rapidly reached 80 wt% of CDW after 36–48 h, consistent with classical nitrogen-limitation-induced PHB synthesis. In contrast, increasing nitrogen availability (2–5 g/L) markedly suppressed PHB accumulation (Fig. 1B), confirming nitrogen availability as a dominant regulator of polymer formation. Across the tested nitrogen levels, 2 g/L urea consistently supported the highest CDW in both strains *H. bluephagenesis* WZY278 and WZY278*ΔphaZ1*23, whereas nitrogen limitation (0.5 g/L) or excess (5 g/L) nitrogen reduced cell growth with reduced CDW (Figs. s1-s2). True cell mass (TCM) analysis further confirmed that biomass peaked at 2 g/L urea (Fig. s3), indicating that carbon-nitrogen balance rather than *phaZ*-dependent turnover governs PHB accumulation. Accordingly, 2 g/L urea with 40 g/L glucose was selected as the optimized condition for all subsequent shake-flask studies unless otherwise stated.

Further improvements for PHB production will likely require intervention in other metabolic pathways. We hypothesized that deletion on *phaZ* could enhance PHB retention by reducing intracellular mobilization. Four *phaZ* homologs (*phaZ*1–*phaZ*4) were identified in the genome of *H. bluephagenesis*, and a triple-deleted strain, namely, *H. bluephagenesis* 278Δ*phaZ*123, was constructed using the CRISPR/AID system [31]. Although this strain accumulated more PHB than the wild type at certain nitrogen levels (Fig. 1C), it exhibited reduced CDW, and neither individual nor combined *phaZ* deletions (TD08) produced substantial improvements in PHB content or CDW compared with the parent strain (Fig. 1D-E). These findings agree with earlier data showing that *H. bluephagenesis* does not significantly elevate PHB via *phaZ* disruption alone. But contrast with studies in *Pseudomonas putida* [32], where deletion of a single *phaZ* gene increased medium-chain-length PHA content by approximately 20 wt%. Collectively, the results demonstrated that *phaZ*-mediated depolymerization is not the primary bottleneck under nitrogen rich conditions, and that further improvement of PHB synthesis requires targeting regulation on upstream metabolic nodes.

### 3.2 Disruption of *ilvA* triggers pseudo-nitrogen starvation for enhanced PHB accumulation under nitrogen rich condition

**Fig. 2:**
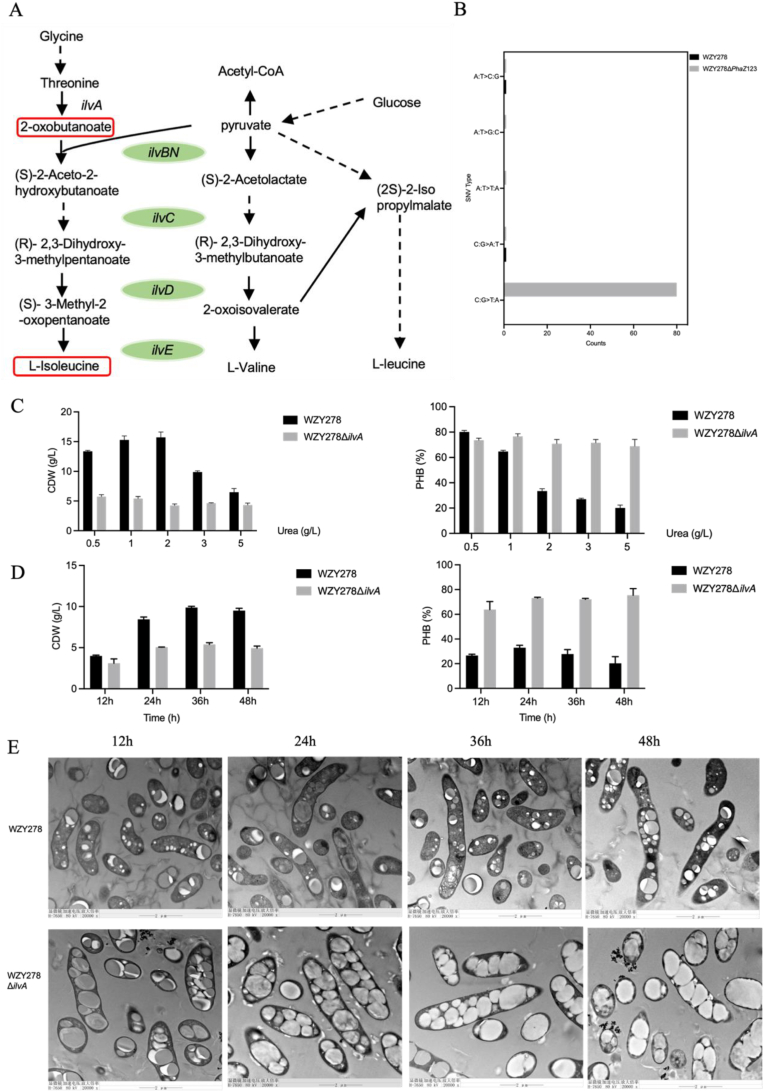
Effects of *ilvA* deletion on branched-chain amino acid metabolism and PHB synthesis by related *H. bluephagenesis* mutants grown under various nitrogen levels, respectively. (A) The branched-chain amino acid synthetic pathway in *H. bluephagenesis*, highlighting the *ilvA*-catalyzed conversion within the isoleucine branch and its position relative to upstream acetyl-CoA and pyruvate flux. (B) Distribution and frequency of single-nucleotide variants identified in *H. bluephagenesis* WZY278 and the *ilvA*-deleted mutant. (C) Comparative CDW and PHB synthesis by *H. bluephagenesis* WZY278 and its *ilvA*-deleted strain grown on graded urea concentrations during shake-flask cultivation. (D) Time-course comparison of CDW and PHB accumulation between *H. bluephagenesis* WZY278 and its *ilvA*-deficient strain over a 48-h growth. (E) TEM visualization illustrating intracellular PHB granule morphology in *H. bluephagenesis* WZY278 and the *ilvA*-deleted mutant grown at 12, 24, 36, and 48 h, respectively.

Although previous studies showed that deleting *phaZ* genes did not enhance PHB under nitrogen limitation or excess, the triple-deleted *H. bluephagenesis* WZY278Δ*phaZ1*23 nonetheless exhibited unexpectedly high PHB accumulation. This unexpected observation prompted further investigation into the genetic basis of the phenotype. We hypothesized that the phenotype may be associated with unintended off-target edited mutations introduced during strain construction by CRISPR/Cas9-AID base-editing system, to identify the potential genetic cause, whole-genome sequencing was performed, revealing 86 single-nucleotide variants (SNVs) relative to the parental strain, most of which were C·G→T·A transitions characteristic of the Cas9-AID base editor (Fig. 2B). Several SNVs introduced premature stop codons in genes associated with stress response or carbon-nitrogen metabolism. One candidate, *csbD* encoding a σ^B^-regulated stress-response protein [33], its Δ*csbD* mutant showed no difference from the wild type in PHB turnover or cell growth (Fig. s4). To determine whether the phenotype was related to nitrogen source composition, we further supplemented the medium with complex nitrogen sources rich in amino acids and small peptides. Under these conditions, addition of yeast extract or tryptone eliminated the PHB difference between *H. bluephagenesis* WZY278Δ*phaZ*123 and the parental strain (Figs. s5–s6), suggesting that the altered PHB synthesis is more likely associated with amino-acid metabolism rather than with nonspecific physiological stresses.

Based on the above analysis, metabolism-related genes were further screened, and it was found that a premature stop codon was also detected in *ilvA* encoding threonine deaminase, the first committed step in BCAA biosynthesis [21] (Fig. 2A). To test its role, a clean Δ*ilvA* mutant was constructed. As predicted, Δ*ilvA* in *H. bluephagenesis* exhibited strongly elevated PHB accumulation but markedly reduced biomass under nitrogen-rich conditions (Fig. 2C). Supplementation studies further supported this phenotype: 0.5 g/L isoleucine restored wild-type growth and PHB synthesis, whereas higher levels on isoleucine inhibited growth (Fig. s7). In contrast, α-ketobutyrate supplementation lowered both CDW and PHB (Fig. s8), indicating that precursor accumulation imposes a metabolic burden rather than relieving the bottleneck [34].

Over the nitrogen concentration range, Δ*ilvA* in *H. bluephagenesis* showed complete decoupling of PHB from nitrogen repression: PHB increased rapidly within 24 h and remained high, whereas biomass plateaued early (Fig. 2D). Wild-type *H. bluephagenesis* cells showed delayed PHB accumulation. TEM analysis corroborated these differences (Fig. 2E): Δ*ilvA* cells contained one or two large and compact PHB granules occupying most of the cytoplasm space, whereas wild-type cells displayed multiple smaller granules; Δ*ilvA* in *H. bluephagenesis* also showed thinner envelopes and fewer dividing cells.

To determine whether polymer properties were altered, PHB molecular weights were compared. Δ*ilvA* in *H. bluephagenesis* produced slightly higher molecular weight PHB (M_n_ 19.58 vs. 16.90 kDa; M_w_ 39.49 vs. 36.00 kDa), yet overall distributions remained similar (Fig. s9). These results suggest that deletion of *ilvA* did not markedly affect the final degree of PHB polymerization. However, molecular weights alone are insufficient to directly infer whether the kinetic parameters of PhaC-catalyzed polymerization have been altered [35]. Instead, the deletion of *ilvA* has only a limited effect on the molecular weight distribution of PHB, the elevated PHB results may come from increased precursor availability.

Together, these results identified *ilvA* as the key genetic determinant underlying the Δ*phaZ*123 phenotype and established that loss of *ilvA* reshaped nitrogen and BCAA metabolism, leading to high PHB accumulation but reduced biomass under nitrogen-rich conditions.

### 3.3 Deletion on *ilvA* rewires nitrogen signaling and drives PHB overproduction

**Fig. 3:**
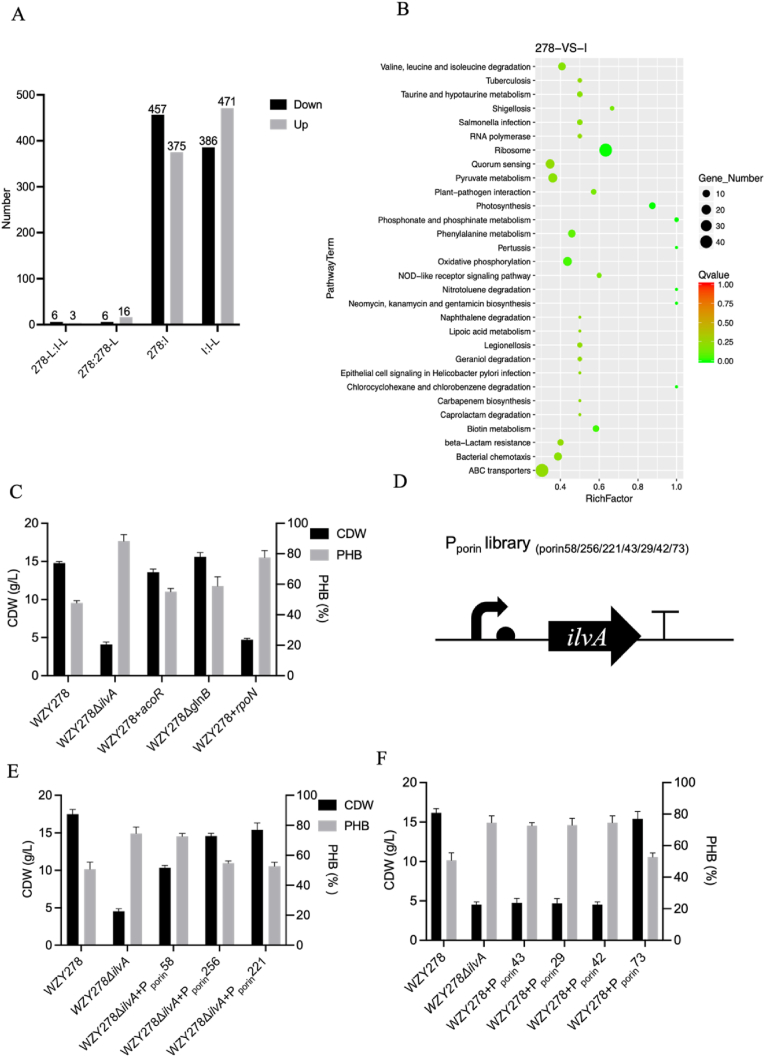
Transcriptomic responses and promoter-controlled *ilvA* re-expression strategies for *H. bluephagenesis*. (A) Summary of upregulated and downregulated genes identified in *H. bluephagenesis* WZY278 and its *ilvA*-deficient mutant across comparative transcriptomic groups. (B) KEGG enrichment analysis comparison illustrates significantly affected metabolic pathways in the WZY278 versus its *ilvA*-deleted mutant, with pathway-level enrichment factors and gene counts. (C) Comparative CDW and PHB content of *H. bluephagenesis* strains harboring deletions or overexpression constructs of Δ*ilvA*, *acoR*, *glnB* and *rpoN*, respectively, during shake-flask cultivation. (D) Schematic representation of the porin promoter library employed to modulate *ilvA* transcriptional output using constitutive promoters of varying strengths. (E) Evaluation of CDW and PHB accumulation by *H. bluephagenesis* strains expressing *ilvA* under different porin promoters (porin58, porin256, porin221) introduced into the Δ*ilvA* mutants, respectively. (F) Comparative CDW and PHB production of *H. bluephagenesis* WZY278 strains overexpressing *ilvA* under different porin promoters (porin43, porin29, porin42, and porin73), enabling graded levels of *ilvA* expression.

To elucidate the metabolic consequences of *ilvA* deletion under nitrogen-rich conditions, transcriptomic profiling was performed for *H. bluephagenesis* WZY278 (278) and its *ilvA* mutant (I), with or without 0.5 g/L isoleucine (278-L, I-L). Cultures were sampled at 5 g/L urea to maximize nitrogen-responsive transcriptional differences. Isoleucine supplementation produced only minor transcriptional and phenotypic changes (Fig. 3A), so subsequent analyses focused on *H. bluephagenesis* WZY278 versus *H. bluephagenesis* WZY278Δ*ilvA*. In total, 832 genes were differentially expressed, including 375 upregulated and 457 downregulated genes (Fig. 3A).

KEGG enrichment indicated broad transcriptional reprogramming in *H. bluephagenesis* WZY278Δ*ilvA*, with significant representation of pathways involved in ribosome function, RNA polymerase, ABC transporters, quorum sensing, bacterial chemotaxis, and central carbon metabolism (pyruvate metabolism, oxidative phosphorylation), as well as BCAA biosynthesis and degradation (Fig. 3B).

At the individual gene level, multiple nitrogen-assimilation genes were induced. The σ^54^ factor *rpoN* and its downstream target gene *glnA* (glutamine synthetase) were significantly upregulated, together with BCAA biosynthetic genes *ilvBN*, *ilvC*, *ilvD*, and *ilvE* [36].

To investigate whether these transcriptional alterations influence PHB synthesis, regulatory genes involved in nitrogen assimilation or *acoR* [37], *glnB* [38], and *rpoN* were individually disrupted or overexpressed following the transcriptional trends observed in the RNA-seq data. All strains exhibited different growth comparable to that of the wild type (Fig. 3C), with PHB contents also varied: the Δ*ilvA* mutant retained the highest PHB levels, whereas *acoR* overexpression and *glnB* deletion did not increase PHB accumulation under nitrogen-rich conditions, showing levels comparable to the wild type (Fig. 3C).

In the *ilvA*-deleted *Halomonas* strains, *ilvA* was overexpressed using a panel of constitutive porin promoters of different strengths, respectively [39]. When the promoter activity was sufficiently high (e.g., P_porin58_ driving *ilvA* expression), the strain exhibited a lower cell dry weight and higher PHB accumulation compared with the P_porin256_- and P_porin221_-driven constructs (Fig. 3E). This observation led us to hypothesize that strong *ilvA* expression may similarly trigger a “low-CDW/high-PHB” phenotype. Consistently, overexpressing *ilvA* in the wildtype *H. bluephagenesis* WZY278 confirmed this pattern: strong porin-promoter-driven *ilvA* expression resulted in the same low-CDW/high-PHB phenotype (Fig. 3F). Across *H. bluephagenesis* strains with altered *ilvA* expression, a non-monotonic phenotype was observed: both strong overexpression and strong repression led to reduced CDW but elevated PHB (Fig. 3E-F). However, promoter and RBS refactoring failed to identify an intermediate “sweet spot” that increased PHB without impairing growth (Figs. s3–s4).

### 3.4 Fine-tuning of *ilvA* boosts PHB synthesis under high nitrogen without growth penalty

**Fig. 4:**
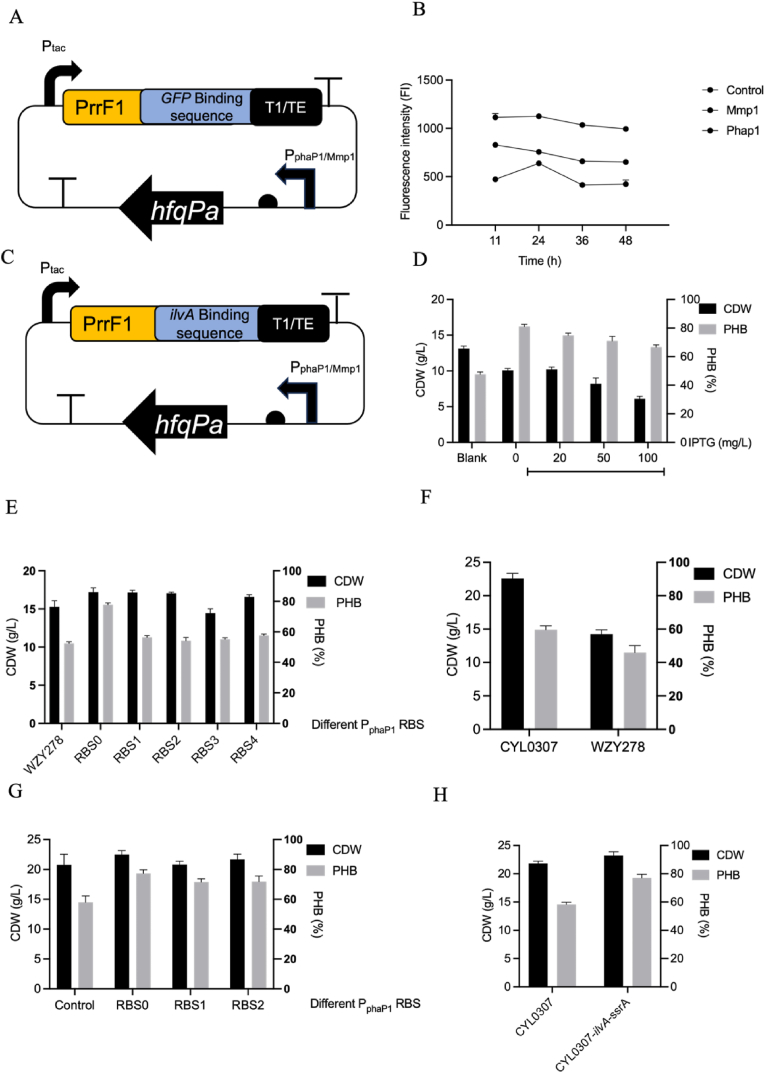
Multi-layer control of *ilvA* enables tunable decoupling between cell growth and PHB accumulation by *H. bluephagenesis*. (A) Constructs of the sRNA-Hfq module targeting the GFP-binding sequence controlled by P_tac_ equipped with the PrrF1 scaffold and transcriptional terminator, respectively. (B) Time-course fluorescence profiles of reporter constructs harboring the GFP-binding sequence, compared to the control, P_Mmp1_-driven, and P_*phaP1*_-driven expression modules. (C) Schematic illustration of the sRNA-Hfq module redesigned to target the *ilvA*-binding sequence for post-transcriptional repression. (D) Comparative CDW and PHB synthesis by *H. bluephagenesis* strains expressing *ilvA*-targeting sRNA under varying IPTG induction strengths. (E) Evaluation of CDW and PHB accumulation by *H. bluephagenesis* strains expressing the sRNA module under different RBS strength promoters (*phaP1* variants), enabling graded *ilvA* repression, respectively. (F) Comparative CDW and PHB production by *H. bluephagenesis* CYL0307 and the parental *H. bluephagenesis* WZY278 under identical cultivation conditions, respectively. (G) CDW and PHB synthesis by *H. bluephagenesis* CYL0307 expressing sRNA modules paired with distinct *phaP1* promoters (RBS0-RBS2), illustrating promoter-dependent tuning of sRNA output. (H) Comparative CDW and PHB accumulation by *H. bluephagenesis* CYL0307 and a derivative harboring *ilvA*-sRNA-*ssrA* repression, highlighting the effect of strengthened post-transcriptional silencing.

Two orthogonal and dose-controllable strategies were adopted to decouple growth from PHB gains: (i) small RNA (sRNA)-mediated translational knockdown and (ii) SspB-assisted proteolysis of *ilvA*. To establish a tunable sRNA system, we first assessed how promoter strength influences Hfq-dependent repression using a GFP reporter. With a constant sRNA scaffold, Hfq controlled by P_*phaP1*_ or P_Mmp1_ markedly reduced fluorescence relative to that of the control strain (Fig. 4B). P_Mmp1_, under full induction of IPTG [40], produced the strongest repression. The *phaP1* promoter constitutes a product-responsive regulatory architecture in which repression is relieved as PHB accumulated. PhaR represses the *phaP1* promoter in the absence of PHB, whereas PHB granule formation sequesters PhaR away from DNA, thereby relieving repression and activating P_*phaP1*_-driven gene expression [41]. The inducer-free P_*phaP1*_ was therefore selected to minimize metabolic burden.

To test whether sRNA repression could redirect flux toward PHB synthesis in a growth-compatible manner, an IPTG/Mmp1-driven *ilvA*-targeting sRNA module was constructed. Although induction at 20–50 mg/L IPTG modestly increased PHB, CDW was consistently impaired (Fig. 4D), indicating that the Mmp1-IPTG circuit was not scalable. Consequently, we placed the *ilvA*-sRNA under P_*phaP1*_, Systematic variation of the upstream RBS further expanded the dynamic range. The strongest RBS variant RBS0 avoided growth defects and slightly increased CDW while achieving 78% PHB synthesis (Fig. 4E).

We also compared *H. bluephagenesis* WZY278 and CYL0307 under nitrogen-rich conditions. Owing to its enlarged morphology, *H. bluephagenesis* CYL0307 exhibited higher CDW and PHB than *H. bluephagenesis* WZY278 (Fig. 4F). The sRNA-mediated modulation in *H. bluephagenesis* CYL0307 produced only modest effects; among the constructs, the P_*phaP1*_-targeting sRNA generated the strongest increase in CDW and PHB (Fig. 4G), suggesting that sRNA-based regulation can influence PHB synthesis even in enlarged cells.

Finally, SsrA-mediated protein degradation of IlvA was also evaluated. To examine whether proteolysis-based regulation could provide tunable control of IlvA abundance, we attempted to construct additional SsrA-tag variants with different degradation strengths. However, only the SsrA-21 variant was successfully obtained after multiple attempts. The *H. bluephagenesis* CYL0307-*ilvA*-*ssrA* reached 78% PHB while maintaining CDW comparable to *H. bluephagenesis* CYL0307 (Fig. 4H), demonstrating that targeted proteolysis alone can redirect flux toward PHB synthesis without compromising cell growth. The result indicate that proteolysis-based regulation has the potential to fine-tune enzyme levels, although a broader tunability remains to be established.

Using these two levers: sRNA-mediated repression and SsrA-tag-driven proteolysis, it consistently achieved 20% increase in PHB synthesis (content) under high-nitrogen conditions without detectable biomass loss, contrasting with the growth penalties associated with coarse *ilvA* overexpression or complete repression (Fig. 3F).

To examine how complete *ilvA* deletion reshapes nitrogen metabolism, extracellular abundance of 20 amino acids was quantified using targeted metabolomics. Eighteen amino acids were reliably detected above the quantification limit, and their relative levels exhibited a markedly different pattern between the wild-type *H. bluephagenesis* WZY278 and its Δ*ilvA* mutant (Fig. s12). Across nearly all detected amino acids, *H. bluephagenesis* WZY278Δ*ilvA* showed elevated abundance compared with *H. bluephagenesis* WZY278, indicating a global shift in nitrogen assimilation following loss of threonine deaminase activity. Among all metabolites, valine levels increased from 0.02 g/L to 0.18 g/L in the supernatant and further to 0.35 g/L in whole-cell extracts (Fig s13). Phenylalanine also accumulated significantly in *H. bluephagenesis* WZY278Δ*ilvA*, reaching 0.03 g/L intracellularly, compared with 0.01 g/L in *H. bluephagenesis* WZY278 (Fig s14). Together, these results demonstrated that *ilvA* deletion triggers a substantial reorganization of amino-acid pools, with the strongest effects observed in BCAA related metabolites such as valine, while other amino acids exhibit more moderate but consistent increases.

### 3.5 Growth and PHB production by recombinants *H. bluephagenesis* incubated in 7 L bioreactor

**Fig. 5:**
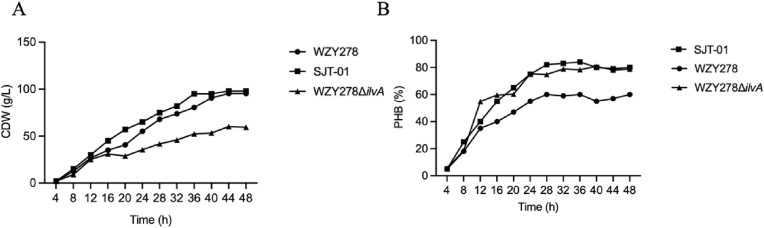
Growth and PHB production by recombinants *H. bluephagenesis* incubated in 7 L bioreactors, respectively. (A) Time-course CDW profiles of *H. bluephagenesis* WZY278, its *ilvA*-deletion strain, and the SJT-01 strain (the sRNA scaffold for inhibiting *ilvA* was knocked in at the G7 site) during growth in a 7 L bioreactor over a period of 48 h. (B) Time-course PHB content of the same three strains cultivated in the 7 L bioreactor, illustrating differences in PHB accumulation dynamics throughout the cultivation processes, respectively.

To evaluate whether the sRNA-mediated repression of *ilvA* remains effective during scale-up, the engineered *H. bluephagenesis* SJT-01 was cultivated in a 7 L bioreactor under nitrogen-rich conditions and compared with both the wildtype *H. bluephagenesis* WZY278 and the *ilvA*-deleted *H. bluephagenesis* WZY278Δ*ilvA*. The sRNA *H. bluephagenesis* SJT-01 exhibited robust growth kinetics, closely matching the CDW trajectory of the wild-type throughout the 48 h growth (Fig. 5A). Final CDW of both strains converged at approximately 95 g/L, demonstrating that sRNA-driven translational repression did not impose a growth penalty at a large scale. In contrast, the *ilvA* deleted *H. bluephagenesis* WZY278Δ*ilvA* showed markedly impaired biomass formation of roughly 60 g/L, consistent with the essential role of finely tuned *ilvA* activity in supporting cell growth under high nitrogen.

Despite maintaining wildtype-like growth, *H. bluephagenesis* SJT-01 achieved substantially higher PHB accumulation. PHB content rapidly increased during the production phase and stabilized between 80%, exceeding the wildtype 60% across the entire culture process. This improvement mirrored the phenotype observed in shake-flask studies, confirming that the sRNA module remains stable and fully functional during growth in bioreactors. Notably, *H. bluephagenesis* SJT-01 also outperformed *H. bluephagenesis* WZY278Δ*ilvA*, which reached high PHB levels only at the cost of severely reduced CDW, highlighting that sRNA knockdown provides a more balanced control of *ilvA* than genomic deletion.

Collectively, these results demonstrated that sRNA-mediated repression of *ilvA* is scalable, genetically stable, and physiologically robust, enabling simultaneous maintenance of biomass formation and enhancement of PHB synthesis under nitrogen-rich conditions. This strategy effectively decouples PHB accumulation from nitrogen limitation without compromising their productivity.

## 4 Discussion and conclusion

This study shows that *ilvA* expression strongly influences the balance between biomass formation and PHB accumulation by *H. bluephagenesis* under nitrogen-rich conditions. The σ^54^ is a well-established bacterial sigma factor that coordinates nitrogen assimilation and utilization of alternative carbon/nitrogen sources, functioning with enhancer-binding proteins to regulate gene expression linked to nitrogen metabolism [42,43]. Demonstrated via deletion, overexpression and promoter-refactoring studies, *ilvA* exhibited a non-monotonic regulatory effect [44]: both excessive and insufficient activity shifted cells toward a “low-CDW/high-PHB” phenotype, suggesting that threonine deaminase occupies a metabolically sensitive position influencing the balance between cellular growth and PHB accumulation (Fig. 3E-F). This bidirectional response demonstrated that *ilvA* activity must be maintained within a relatively narrow functional range, and that conventional transcriptional tuning through promoter or RBS engineering may be insufficient to access a growth-compatible regime supporting both robust biomass formation and high PHB production.

Transcriptomic profiling further clarified how *ilvA* disruption reshapes intracellular physiology (Fig. 3B). Loss of *ilvA* was accompanied by transcriptional changes in several nitrogen metabolism-related pathways, with expression patterns that partially resemble those observed under nitrogen stress conditions [45]. Metabolomic analysis showed that the intracellular levels of most amino acids were unexpectedly elevated although branched-chain amino acid biosynthesis was disrupted in the Δ*ilvA* mutant, suggesting that *ilvA* deletion perturbs nitrogen metabolic homeostasis beyond simply limiting BCAA supply. One possible explanation is that the Δ*ilvA* mutant exhibits a markedly reduced growth rate, which likely decreases cellular demand for protein synthesis and consequently lowers amino-acid consumption, leading to passive intracellular accumulation. In addition, BCAA deficiency may be perceived as a metabolic imbalance signal associated with adjustments in nitrogen assimilation pathways and associated regulatory networks, such as the GS/GOGAT system or σ^54^-mediated regulation. From a systems perspective, these observations suggest that perturbation of *ilvA* disrupts the coordination between nitrogen metabolism and cellular growth, leading to a nitrogen-limitation-like physiological state despite sufficient external nitrogen supply. Reduced biosynthetic demand under slower growth conditions lead to the accumulation of carbon intermediates and reducing equivalents, which can be redirected toward PHB synthesis. In this context, PHB accumulation may function as a metabolic sink that helps rebalance excess carbon flux and redox equivalents even under nitrogen-rich conditions. Collectively, the transcriptome and metabolite data support a model in which *ilvA* functions as a flux-sensitive metabolic node linking BCAA metabolism with nitrogen-associated physiological responses: perturbation of its activity disrupts BCAA entry flux, and may contribute to redistribution of carbon flux and reducing equivalents toward PHB even in nitrogen-rich media (Fig. 2A). These observations point to a possible interaction between BCAA metabolism and nitrogen-responsive regulatory networks.

Given the sharp phenotypic transitions associated with *ilvA* dosage, two orthogonal layers of fine control were integrated to flatten the local control landscape. sRNA-mediated translational repression provided a tunable mechanism for controlling *ilvA* expression. Synthetic small regulatory RNAs have been commonly adopted in metabolic engineering as modular tools for post-transcriptional regulation. While SspB/ClpXP-assisted proteolysis enabled rapid post-translational modulation of protein abundance independent of transcription (Fig. 4H). Because these regulatory layers act at different stages of gene expression, they enable finer tuning of *ilvA* dosage than genomic perturbation alone. This layered control revealed growth-neutral states that increased PHB accumulation by ∼20% under nitrogen sufficiency without reducing CDW (Fig. 4E).

Importantly, these regulatory strategies retained their function during process-scale cultivation. In 7 L fed-batch growth studies, the sRNA regulated strain maintains wildtype-like CDW trajectories (95 g/L), while achieving substantially higher PHB contents of 80% under nitrogen-rich conditions (Fig. 5A-B). This contrasted sharply with the Δ*ilvA* mutant, which attained high PHB fractions only at the cost of severely reduced biomass (60 g/L) (Fig. 5A-B). The scale-up performance demonstrated that sRNA-mediated repression indicated physiological robustness, and process compatibility, underscoring its superiority to genomic deletion for industrial PHB production under nitrogen-rich conditions (Fig. 5A-B). Moreover, the ability to maintain high CDW while elevating PHB directly addresses longstanding challenges in intensifying PHB production without resorting to nutrient limitation.

Although *ilvA* deletion increased intracellular amino-acid pools, absolute titers of valine and phenylalanine remained modest (Figs. s13-s14), indicating that carbon partitioning overwhelmingly favored PHB synthesis rather than amino-acid secretion, suggesting that large-scale co-production of PHB and amino acids is currently limited by export capacity, feedback regulation and intracellular allocation constraints [46]. Future efforts may explore feedback-resistant BCAA-pathway enzymes, transporter engineering to enhance secretion, and *in situ* removal strategies to relieve intracellular bottlenecks. At the systems level, integrating ^13^C fluxomics, proteomics, targeted enzyme assays, and measurements of intracellular nitrogen status indicators, e.g., glutamine/glutamate ratio, will help clarify how *ilvA* perturbation reshapes central carbon-nitrogen metabolism and identify additional branch points amenable to growth-neutral tuning. Integrating multi-omics approaches has become a key strategy for identifying regulatory bottlenecks and optimizing metabolic pathways in engineered microorganisms [47].

In summary, *ilvA* represents a flux-sensitive metabolic node linking BCAA metabolism with nitrogen-associated physiological responses and carbon partitioning in *H. bluephagenesis*. Tunable multi-layer regulation combining sRNA-mediated translational repression and SspB/ClpXP-assisted proteolysis enabled partial decoupling of growth from PHB accumulation under nitrogen-rich conditions. These provide a practical strategy for improving PHB production without sacrificing biomass formation, and illustrate how multi-layer control can be applied to fine-tune flux-sensitive metabolic nodes in industrial microorganisms.

## CRediT authorship contribution statement

**Junting Sheng:** Writing – original draft, Visualization, Methodology, Investigation. **Yuying Guan:** Methodology. **Jiale Wang:** Methodology. **Xu Liu:** Methodology. **Zhenghao Xu:** Methodology. **Yiling Chen:** Methodology. **Fuqing Wu:** Methodology. **Guo-Qiang Chen:** Writing – review & editing, Supervision, Funding acquisition, Conceptualization.

## Declaration of competing interest

The author Guo-Qiang Chen is the Editor-in-Chief for Synthetic and Systems Biotechnology and was not involved in the editorial review or the decision to publish this article. The authors declare the following financial interests which may be considered as potential competing interests: the research project is funded by Beijing PhaBuilder Biotechnology Co. Ltd.

## Data Availability

Data will be made available on request.
