# Supplementary material for: Modulating ilvA encoding threonine deaminase for balanced growth and PHB synthesis by Halomonas grown in rich nitrogen source
Source: Synth Syst Biotechnol. 2026 Apr 16;14:89–100. doi: 10.1016/j.synbio.2026.03.022 (PMC13101641; doi:10.1016/j.synbio.2026.03.022)
Supplement: Multimedia component 1 [file mmc1.docx]

**Supplementary Information**

**Table S1. Strains and Plasmids**

| **Strains** | **Brief Description** | **References/Sources** |
| --- | --- | --- |
| *Escherichia coli S17-1* pir | Harboring the *tra* genes on the chromosome, used for conjugation and plasmid construction. | [1] |
| *Halomonas bluepahgenesis* TD01 | Wild-type strain, used for PHA productions. | Lab stocks |
| TD08 | *Halomonas bluephagenesis* TD01 derivate, knockout of three *phaZ* gene by CRISPR/Cas9. | Lab stocks |
| WZY278 | *Halomonas bluephagenesis* TD01 derivate, knockout of *phaP1*, *lpxM*, *lpxL* gene. | Lab stocks |
| WZY278Δ*phaZ1*23 | *Halomonas bluephagenesis* WZY278 derivate, knockout of the three *phaZ* gene by CRISPR/Cas9 AID. | Lab stocks |
| WZY278Δ*ilvA* | *Halomonas bluephagenesis* WZY278 derivate, knockout of the native *ilvA* gene. | This study |
| WZY278Δ*phaZ1*-WZY278 Δ*phaZ4* | *Halomonas bluephagenesis* WZY278 derivate, knockout of the native *phaZ* (1-4) gene | This study |
| WZY278Δg*lnB* | *Halomonas bluephagenesis* WZY278 derivate, knockout of the native *glnB* gene | This study |
| CYL0307 | TD01 derivate with pHbPBC*-*Pporin-*phaABRe, ΔphaP1, ΔsspB, Δ*enp *mreB-ssrA21* | Lab stocks |
| CYL0307-*ilvA-ssrA* | CYL0307 derivate with *ilvA*-*ssrA*21 on genome | This study |
| SJT-01 | WZY278 derivate, the sRNA scaffold for inhibiting *ilvA* was knocked in at the G7 site | This study |
| **Plasmids** |  |  |
| pSEVA321 | A broad-host medium copy-number expression vector, *oriT*, Cm^R^. | [2] |
| pSEVA341 | A broad-host high copy-number expression vector, *oriT*, KmR and SpR. | [2] |
| pQ08 | pSEVA321 derivate, *S. pyogenes* cas9, Cm^R^ | [3] |
| pSJT1- pSJT7 | pSEVA321 derivate, overexpressing the *ilvA* gene driven by different P_porin_ (P_porin58/256/221/43/29/42/73_) | This study |
| pSJT8- pSJT9 | pSEVA321 derivate, overexpressing the *acoR* and *rpoN* gene driven by P_porin58_ | This study |
| pSJT10-pSJT14 | pSEVA321 derivate, with the sRNA scaffold for inhibiting *ilvA* expression by P_phaP1_ with different RBS (RBS0-4) | This study |
| pSJT15 | pSEVA321 derivate, with the sRNA scaffold for inhibiting *ilvA* expression by P_Mmp1_ | This study |
| pSJT16- pSJT17 | pSEVA321 derivate, with the sRNA scaffold for inhibiting GFP expression by different promoters (P_phaP1_ /P_Mmp1_) | This study |
| pSJT18 | pSEVA321 derivate, overexpressing *ilvA* gene driven by P_Mmp1_ | This study |
| pSJT19 | pSEVA321 derivate, empty plasmid with P_Mmp1_ as control group | This study |
| pSJT20-22 | pSEVA341 derivate, with different gRNA to knock out different genes (*acoR, ilvA, csbD*) | This study |
| pSJT23-26 | pSEVA341 derivate, with different gRNA to knock out different *phaZ* genes (*phaZ*1-*phaZ*4) | This study |

|  |  |  |
| --- | --- | --- |

**Table S2. The DNA sequences of different RBS.**

| RBS Number | | DNA sequence |
| --- | --- | --- |
| RBS 0 | | CTCACTTAAGGAGATTATGTG |
| RBS 1 | | CTCACTTAAGGGGATTATGTG |
| RBS 2 | CTCACTTAGTAGGATTATGTG | |
| RBS 3 | CTCACTTATCGGGATTATGTG | |
| RBS 4 | CTCACTTAGAGGTATTATGTG | |


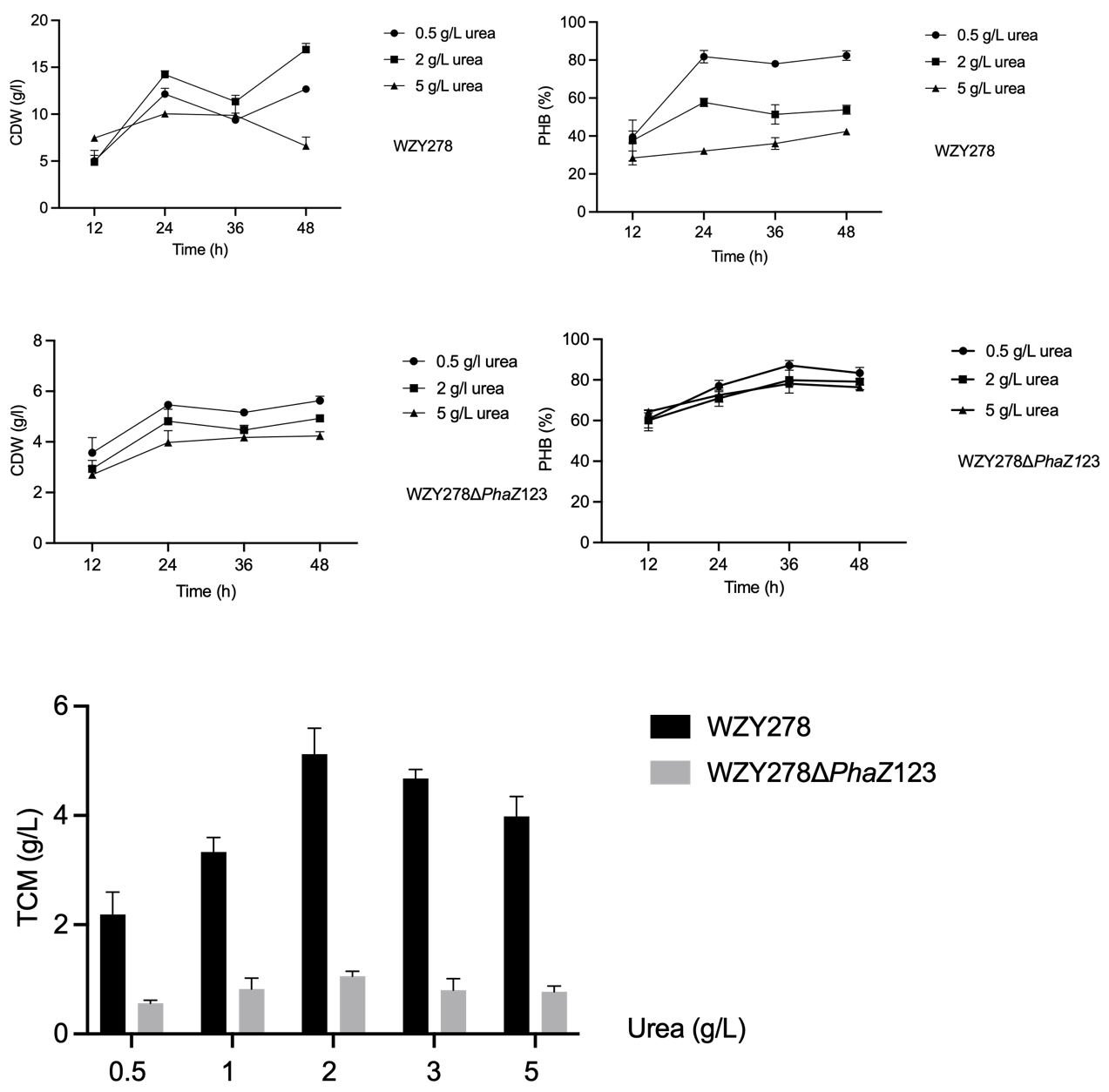


Figures s1-s3. Effects of nitrogen availability on biomass formation and PHB accumulation by wild-type *H. bluephagenesis* WZY278 and the triple *phaZ*-deleted *H. bluephagenesis* WZY278Δ*phaZ*123.

(s1) Growth (CDW) and PHB synthesis (content) of *H. bluephagenesis* WZY278 cultivated under five urea concentrations (0.5, 1, 2, 3 and 5 g/L) during the cultivation, respectively. (s2) Growth (CDW) and PHB synthesis (content) of the triple *phaZ*-deleted *H. bluephagenesis* WZY278Δ*phaZ*123 under the same urea concentrations and cultivation conditions, respectively. (s3) Comparison of true cell mass (TCM=CDW-PHB) between *H. bluephagenesis* WZY278 and WZY278Δ*phaZ*123 across five urea concentrations, illustrating differences in biomass partitioning under varying nitrogen inputs.


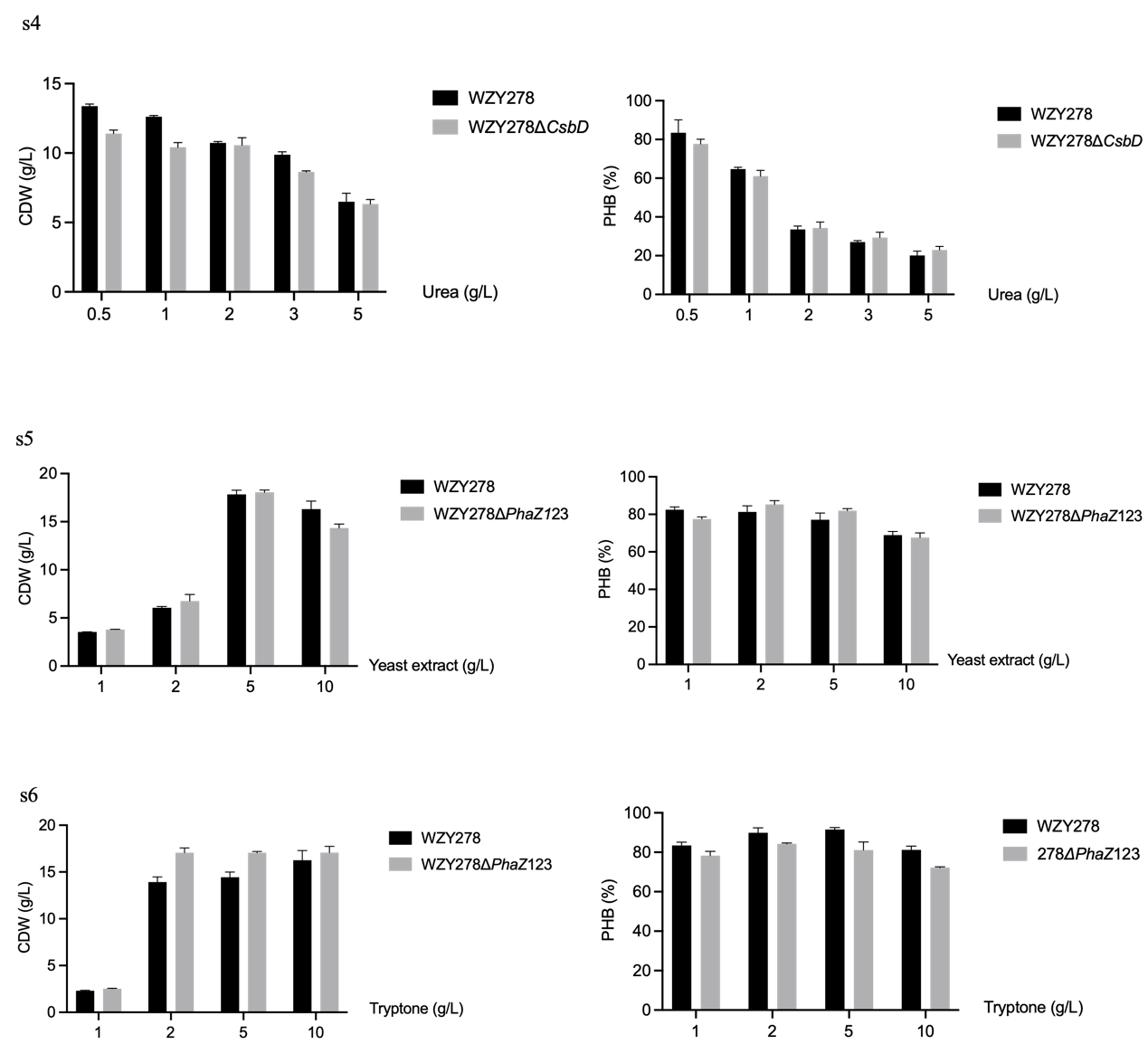


Figures s4-s6. Effects of urea nitrogen and complex nitrogen sources on growth (CDW) and PHB accumulation by *H. bluephagenesis* WZY278Δ*csbD* and 278Δ*phaZ*123.

(s4) Comparative CDW and PHB production by *H. bluephagenesis* WZY278 and WZY278Δ*csbD* grown on various urea concentrations (0.5-5 g/L). (s5) Assessment of CDW and PHB synthesis by *H. bluephagenesis* WZY278 and the triple *phaZ*-deleted *H. bluephagenesis* WZY 278Δ*phaZ*123 cultivated with increasing yeast extract concentrations. (s6) Evaluation of CDW and PHB accumulation by *H. bluephagenesis* WZY278 and WZY278Δ*phaZ*123 cultivated in four tryptone concentrations, respectively.


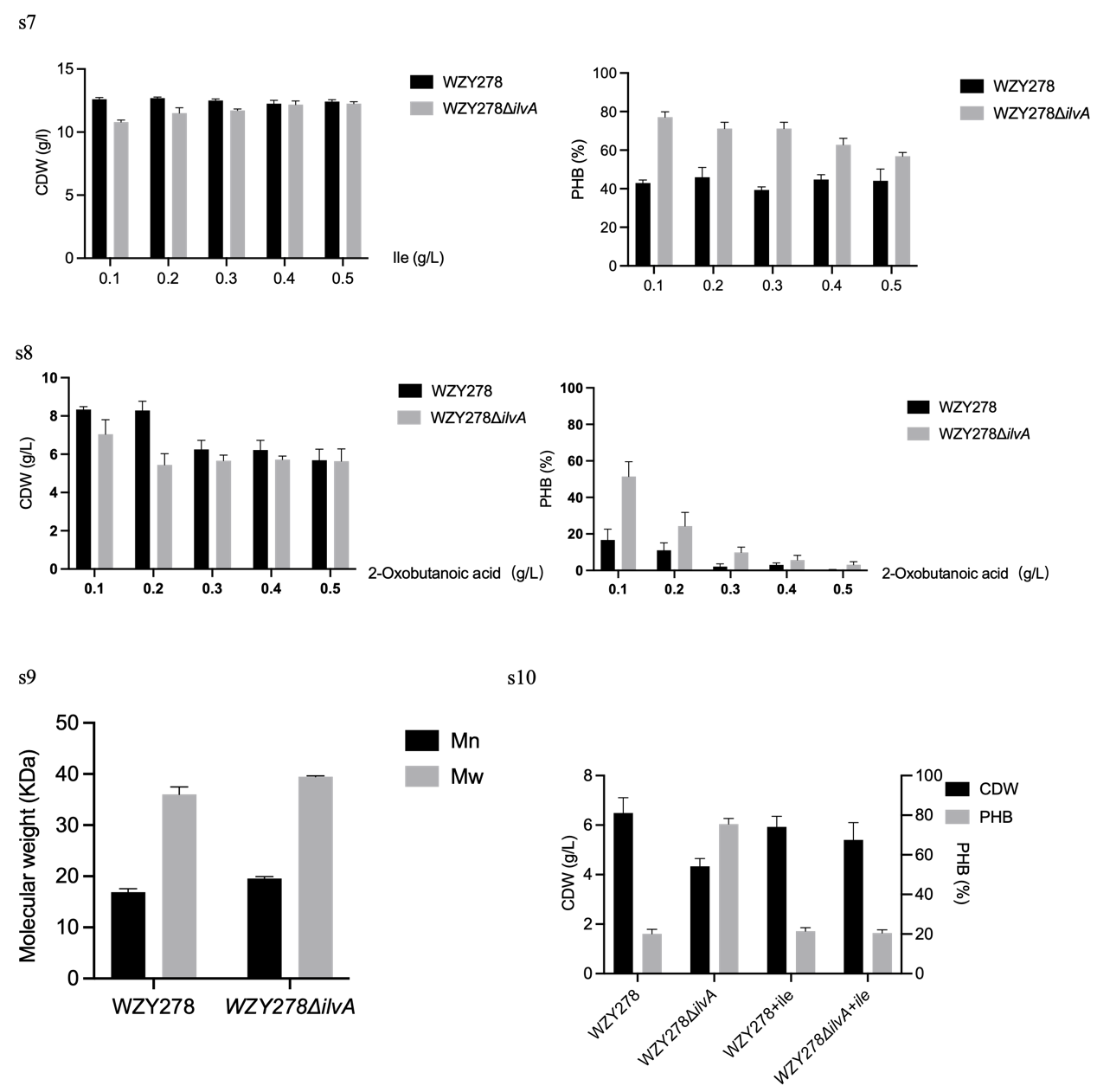


Figures s7-s10. Effects of isoleucine and 2-oxobutanoic acid on growth and PHB synthesis by *H. bluephagenesis* engineered strains.

(s7) Impact of isoleucine supplementation on CDW and PHB synthesis by *H. bluephagenesis* WZY278 and WZY278Δ*ilvA*, respectively. PHB accumulation decreased progressively with increasing isoleucine concentrations, while CDW remained largely stable in both strains. (s8) Effects of 2-oxobutanoic acid on CDW and PHB synthesis by *H. bluephagenesis* WZY278 and WZY278Δ*ilvA*. Increasing 2-oxobutanoic acid concentrations (0.1-0.5 g/L) resulted in a dose-dependent inhibition on PHB synthesis, accompanied by a reduction in CDW. (s9) The inhibitory effect was more pronounced in the *ilvA*-deficient *H. bluephagenesis*. Molecular weight distribution of PHB produced by *H. bluephagenesis* WZY278 and WZY278Δ*ilvA*. Both number-average (M_n_) and weight-average (M_w_) molecular weights were comparable between the two strains, indicating that *ilvA* deletion does not alter polymer chain length. (s10) Growth and PHB phenotypes of RNA-seq samples cultured at 5 g/L urea.


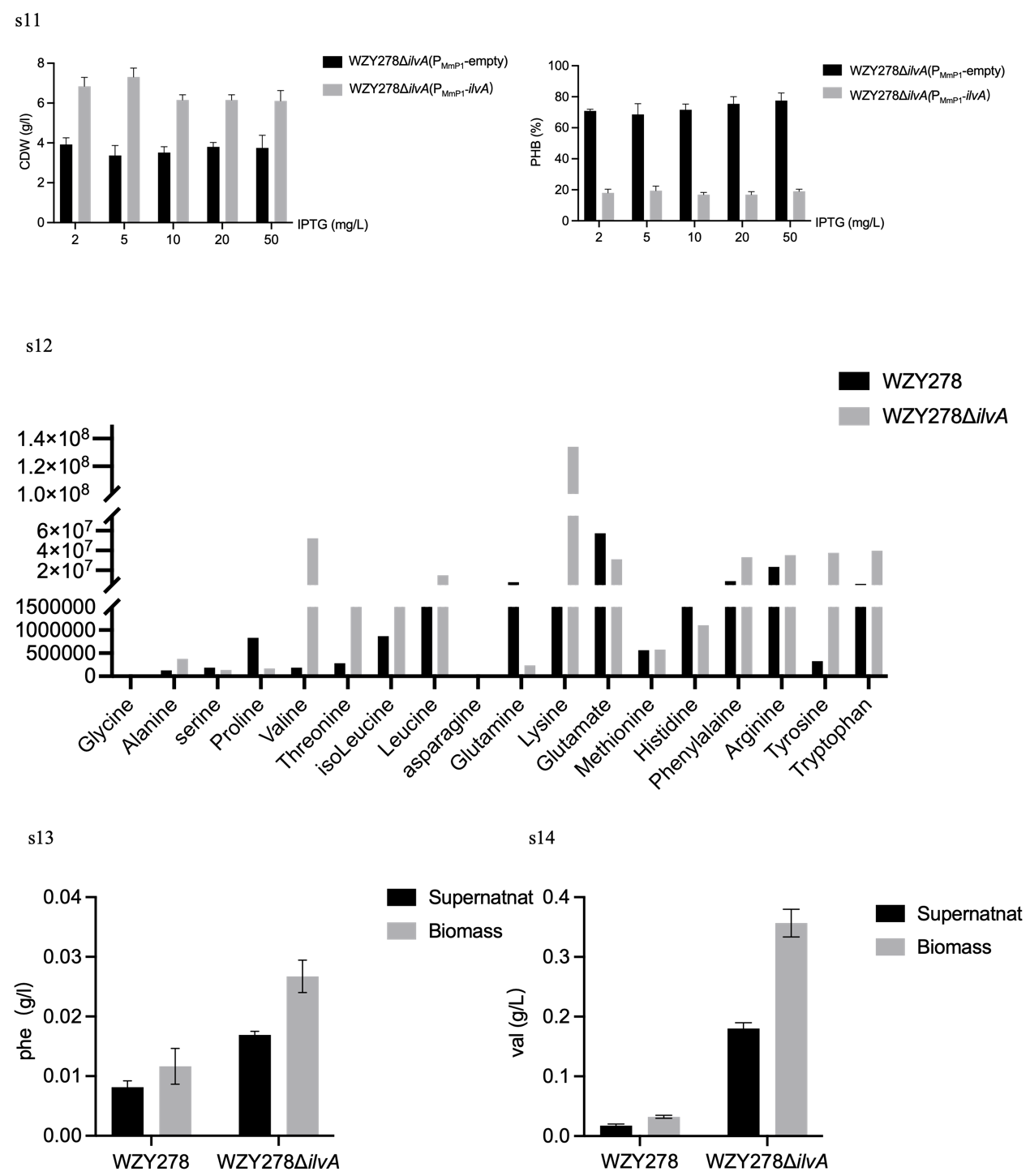


Figures s11-s14. Functional impacts of *ilvA* regulation on coupling growth–production of PHB and amino-acid metabolism.

(s11) IPTG-dependent *ilvA* induction reduces PHB accumulation by *H. bluephagenesis* WZY278Δ*ilvA*. This demonstrates that transcriptional up-regulation of *ilvA* cannot decouple biomass formation from PHB synthesis under nitrogen-rich conditions. (s12) Intracellular amino-acid profiles of *H. bluephagenesis* WZY278 and *H. bluephagenesis* WZY278Δ*ilvA*. Loss of *ilvA* broadly reshaped branched-chain amino-acid pools. (s13-s14) Valine and phenylalanine accumulation by *H. bluephagenesis* WZY278 and WZY 278Δ*ilvA*, including extracellular and intracellular, respectively.

**References**

[1] R. Simon, U. Priefer, A. Pühler, A broad host range mobilization system for *in vivo* genetic engineering: Transposon mutagenesis in gram-negative bacteria, Nat. Biotechnol. 1 (1983) 784-791. <https://doi.org/10.1038/nbt1183-784>.

[2] E. Martínez-García, T. Aparicio, A. Goñi-Moreno, S. Fraile, V. de Lorenzo, SEVA 2.0: An update of the Standard European Vector Architecture for de-/re-construction of bacterial functionalities, Nucleic Acids Res. 43 (2015) D1183-D1189. <https://doi.org/10.1093/nar/gku1114>.

[3] Q. Qin, C. Ling, Y. Zhao, T. Yang, J. Yin, Y. Guo, G.Q. Chen, CRISPR/Cas9 editing genome of extremophile *Halomonas* spp., Metab. Eng. 47 (2018) 219-229. <https://doi.org/10.1016/j.ymben.2018.03.018>.
